# Evaluation of the Quality and Readability of ChatGPT Responses to Toothache Queries: A Study Based on Google Trends

**DOI:** 10.7759/cureus.96436

**Published:** 2025-11-09

**Authors:** Duygu Degirmencioglu, Ayse Nur Temel

**Affiliations:** 1 Endodontics, Istanbul Medipol University, Istanbul, TUR

**Keywords:** chatgpt, cross-sectional study, dental pain, ehealth literacy, eqip, google trends, health communication, readability, smog, toothache

## Abstract

Background

Conversational AI tools such as ChatGPT are increasingly used for health information seeking. While their popularity continues to grow, little is known about the readability and quality of their outputs in dental contexts, particularly for toothache, one of the most common oral health complaints.

Objective

This study aimed to evaluate the quality and readability of ChatGPT’s responses to frequently searched toothache-related queries and to conceptually compare them with information available from top-ranked traditional websites.

Methods

In this cross-sectional study, the 20 most common toothache-related queries were identified using Google Trends (January 2014-January 2024). Each query was posed to ChatGPT (May 2024 version) in independent sessions to avoid contextual bias. Two endodontist raters assessed the quality of responses using the Ensuring Quality Information for Patients (EQIP) tool. Readability was measured using the Flesch Reading Ease, Flesch-Kincaid Grade Level, and Simple Measure of Gobbledygook (SMOG) Index. Interrater reliability was calculated using Cohen’s kappa and the Intraclass Correlation Coefficient (ICC). For comparison, the first 3-5 non-advertising websites retrieved via Google for each query were evaluated using the same instruments.

Results

ChatGPT responses demonstrated high content quality (mean EQIP 85.3 ± 5.2) and moderate readability demands (Flesch Reading Ease 57.9 ± 3.2; Flesch-Kincaid 8.4 ± 0.6; SMOG 7.5 ± 0.4). Interrater reliability was excellent (κ = 0.86; ICC = 0.91). Compared with websites, ChatGPT yielded slightly higher EQIP scores (ΔEQIP + 3.1 on average) but also higher reading grade levels (ΔFKGL + 0.4).

Conclusions

ChatGPT provides responses to toothache-related queries that are comparable, and in some cases superior, in quality to top-ranked, non-advertising traditional health websites retrieved via Google search. However, both ChatGPT and these conventional sources exhibit moderate readability, which may limit accessibility for individuals with low health literacy. Future efforts should focus on simplifying AI-generated and online content to enhance clarity, equity, and effectiveness in digital health communication.

## Introduction

Toothache is one of the most prevalent oral health complaints worldwide, substantially affecting quality of life, productivity, and well-being, and it remains common despite advances in preventive and restorative dentistry [1].

The continued public reliance on online information about toothache reflects a strong need for accessible, accurate, and timely resources, as many individuals turn to the Internet in search of immediate relief before seeking professional care [2].

With the widespread use of the Internet, many individuals seek immediate solutions for tooth pain through digital platforms [3]. Population-level search data indicate that many individuals seek online solutions for toothache [2,4]. This reliance often substitutes professional advice, particularly when access to dentists is constrained. In Turkey, the most frequent toothache queries focused on home remedies and medications, underscoring the need for clear, accessible guidance to prevent unsafe self-management [5].

Online health-seeking behavior is particularly strong in dentistry, where anxiety and urgency often drive patients to search for quick remedies [6,7]. However, the rapid expansion of online content raises concerns about its accuracy, readability, and potential to misinform [8]. Search volumes show seasonal variation, with peaks in spring and summer and sharp increases during periods when dental services were restricted (e.g., during early COVID-19). These patterns reflect fluctuating public information needs over time [5].

Recent advances in AI, especially conversational models such as ChatGPT, provide new opportunities for delivering health information. ChatGPT can generate detailed, conversational responses that mimic human explanations and potentially improve user engagement [9,10]. Yet uncertainties remain regarding the quality and linguistic complexity of such outputs, particularly for audiences with limited health literacy [11].

Health literacy frameworks highlight why readability and quality must be evaluated together. Nutbeam’s model distinguishes functional, interactive, and critical literacy domains, each crucial for effective communication [12,13]. Complementing this, Norman and Skinner’s eHealth Literacy Scale (eHEALS) emphasizes the skills needed to access, understand, and evaluate online health information [14]. Both frameworks suggest that even accurate content may be ineffective if its complexity prevents comprehension [15]. Online interest in toothache is higher in regions with lower socioeconomic status and lower dentist density, suggesting that structural barriers amplify reliance on digital sources and heighten the importance of readable, high-quality content [5].

Against this backdrop of increasing, seasonally variable, and inequity-linked online information seeking, evaluating the readability and patient-facing quality of AI-generated responses has immediate public health relevance. Similar evaluations in other domains, such as spinal cord injury, have shown high information quality but moderate readability; yet the dental field remains unexplored [16-18].

To our knowledge, no study has yet systematically assessed the readability and quality of ChatGPT’s responses to common dental problems or directly compared them with information from conventional online sources. The present study therefore aimed to (1) evaluate the readability and quality of ChatGPT’s responses to the most frequently searched toothache queries identified via Google Trends, and (2) compare these responses with those retrieved from top-ranked traditional websites.

## Materials and methods

Study design

This cross-sectional study analyzed publicly available online data. As no human subjects were involved, formal ethical approval was not required.

Query selection

The 20 most frequently searched queries related to “toothache” between January 2014 and January 2024 were identified using Google Trends. A 10-year window was selected to capture stable patterns and minimize seasonal bias. Only English-language queries were included, while brand-specific or non-English searches were excluded. The use of Google Trends as a digital platform-derived data source is consistent with prior methodological approaches in online behavioral research, which have similarly acknowledged the limitations inherent to platform-based sampling [19].

ChatGPT response collection

Each query was entered into ChatGPT (May 2024 version) in independent chat sessions. Although the data collection took place in July 2024, the ChatGPT interface still displayed the “May 2024 version” label at that time; this version information has been reported accordingly for transparency. Browser data were cleared between sessions to avoid contamination. All responses were collected within a single 48-hour period to minimize model drift and ensure temporal consistency. No role prompts (e.g., “act as a patient”) were used, ensuring that responses reflected ChatGPT’s default output. Each prompt was copied verbatim from Google Trends without modification, and responses were recorded immediately after generation without any manual editing.

Comparator: traditional online sources

For benchmarking, the first 3-5 non-advertisement websites returned by Google for each query were extracted. These texts were evaluated using the same quality and readability instruments applied to ChatGPT outputs, allowing direct comparison across sources.

Quality assessment

The Ensuring Quality Information for Patients (EQIP) tool was applied by two independent evaluators, both board-certified endodontists with clinical and academic experience [20]. Interrater reliability was assessed using Cohen’s kappa (categorical items) and the Intraclass Correlation Coefficient (ICC; continuous scores). Agreement was excellent (κ = 0.86; ICC = 0.91). Discrepancies were resolved through discussion.

Readability analysis

Three validated indices were applied to unedited outputs, including original formatting such as headings and bullet points:

Flesch Reading Ease (FRE) [21],

Flesch-Kincaid Grade Level (FKGL) [22],

Simple Measure of Gobbledygook (SMOG) [23].

Theoretical framework

The study design was informed by two health literacy models. Nutbeam’s framework, which distinguishes functional, interactive, and critical literacy, guided the evaluation of whether outputs supported comprehension (functional), application (interactive), and critical judgment (critical). Norman and Skinner’s eHealth Literacy Scale (eHEALS) emphasizes the skills required to locate, evaluate, and apply online health information [24]. These frameworks justified the dual focus on readability and quality and informed both the choice of evaluation instruments and the interpretation of findings. Nutbeam’s health literacy model, which conceptualizes literacy as comprising functional, interactive, and critical dimensions, has been empirically applied and validated in oral health contexts. For example, Stein L et al. conducted a randomized controlled trial based on Nutbeam’s model within a clinical dental setting, confirming its relevance and reliability for assessing oral health literacy [25]. Although originally developed for general and oral health contexts, Nutbeam’s framework provides a conceptually robust basis for evaluating health literacy in digital environments, including AI-based information such as ChatGPT outputs [24,26].

Ethical considerations and data privacy

All data were aggregated and publicly available, with no personal identifiers. Queries were submitted anonymously, and ChatGPT does not retain user information. Although formal ethical approval was not required, broader ethical concerns were recognized. AI-generated outputs may contain inaccuracies that could encourage self-treatment or delay professional care. Such risks highlight the importance of transparency, safeguards, and professional oversight when integrating AI into patient-facing health communication.

Limitations of study design

This study relied on automated readability indices and expert assessments without user testing, which limits conclusions about real-world comprehension. Moreover, the analysis was restricted to Google Trends queries; future work should include patient-generated content from platforms such as YouTube or Reddit to capture broader information-seeking behaviors. In addition, Google search results are subject to personalization and regional variability, which may influence comparator content despite the use of incognito mode. The study also relied on a single ChatGPT version (May 2024, collected in July 2024), which could introduce minor variability due to potential model updates or parameter adjustments.

## Results

Query trends and distribution

The 20 most frequently searched toothache-related queries identified from Google Trends (January 2014-January 2024) primarily emphasized immediate pain relief and home-based remedies. The three most common queries were “How to stop toothache,” “How to relieve toothache fast,” and “Toothache remedies.”

Immediate pain relief accounted for 55% (11/20) of all queries, followed by home remedies or natural options (25%, 5/20), pharmacological treatments (15%, 3/20), and etiology or causes (5%, 1/20). Overall, 80% of queries focused on immediate or self-manageable solutions, reflecting the urgency and symptom-driven nature of online health information-seeking behavior.

**Figure 1:**
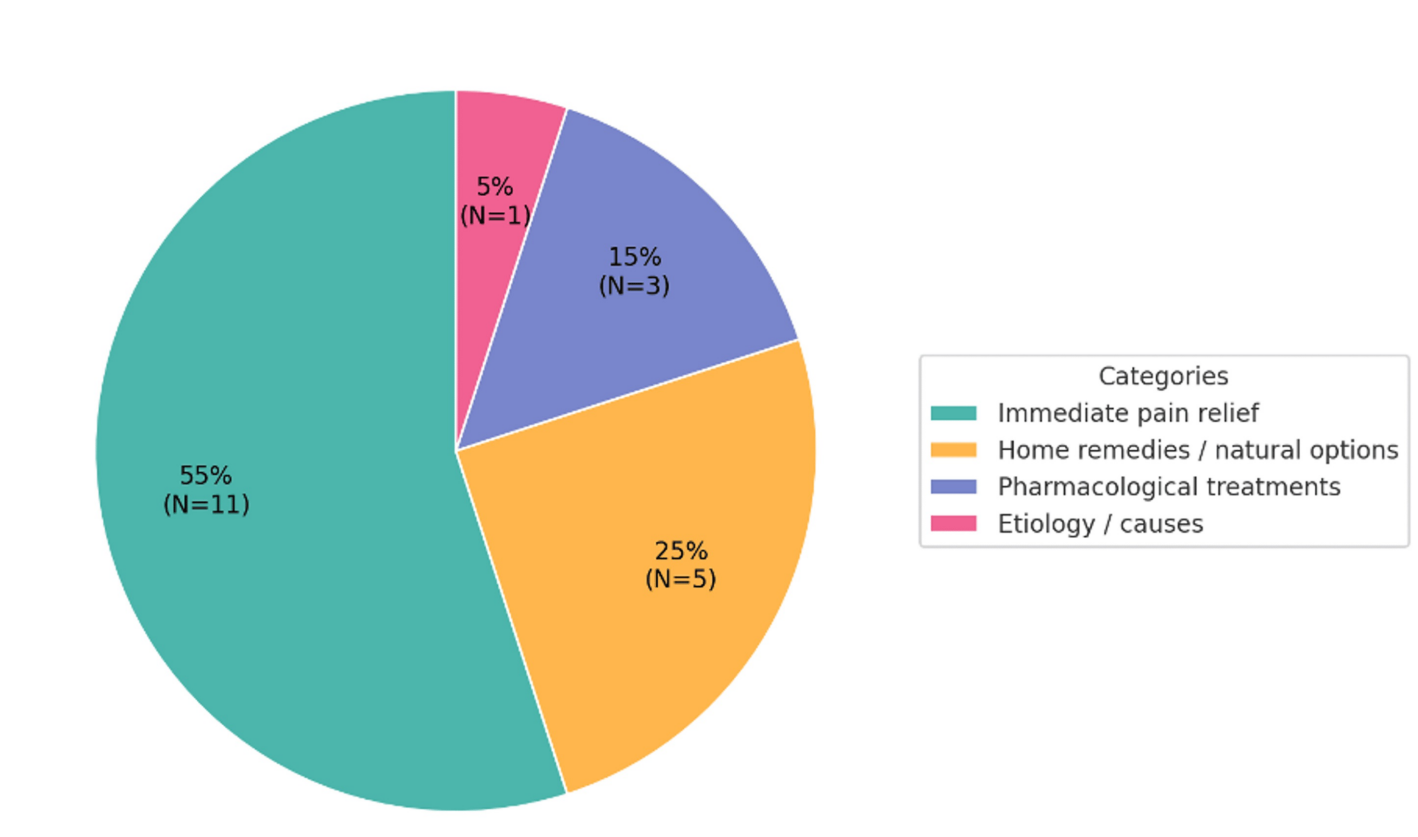
Category distribution (%) and frequency (N) of the 20 most frequently searched toothache-related queries identified from Google Trends (January 2014-January 2024). Most queries focused on immediate pain relief (55%, N = 11), followed by home remedies or natural options (25%, N = 5), pharmacological treatments (15%, N = 3), and etiology or causes (5%, N = 1). These findings suggest that users primarily seek rapid, self-manageable solutions rather than long-term preventive information.

The thematic distribution is summarized in Table 1, with corresponding visual representations shown in Figure 1.

**Table 1:** Distribution of the top 20 toothache-related queries by thematic category. Thematic classification of the 20 most frequently searched toothache-related queries identified from Google Trends (January 2014-January 2024). Queries were grouped into four main categories based on content: immediate pain relief, home remedies or natural options, pharmacological treatments, and etiology or causes.

Category	Number of queries (n = 20)	Examples
Immediate pain relief	11	“How to stop toothache”; “Toothache pain relief”
Home remedies or natural options	5	“Home remedies for toothache”; “Natural remedies”
Pharmacological treatments	3	“Toothache medicine”; “Toothache antibiotics”
Etiology or causes	1	“Toothache causes”

Quality assessment (EQIP Scores)

The EQIP evaluation demonstrated that ChatGPT’s responses were of high quality, with a mean score of 85.3 ± 5.2. This indicates strong performance across clarity, structure, and reliability of information. Interrater agreement was excellent (Cohen’s κ = 0.86; ICC = 0.91), confirming consistency of evaluation.

**Figure 2:**
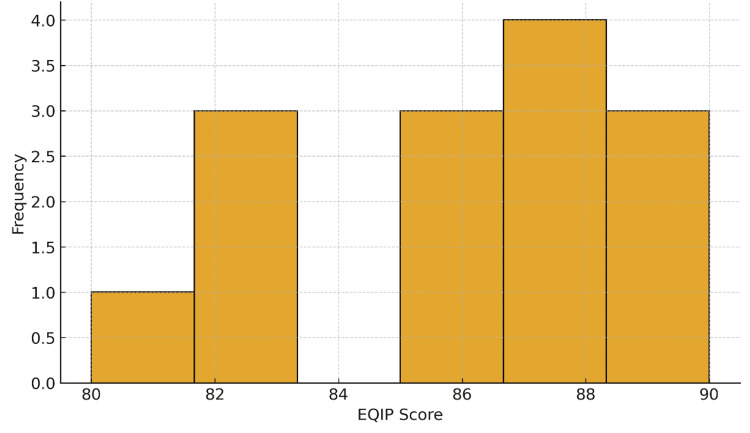
Histogram of EQIP quality scores for ChatGPT responses. Frequency distribution of EQIP scores across the evaluated responses, showing that most responses clustered in the high-quality range, fewer in the moderate range, and very few were rated as low quality. EQIP: Ensuring Quality Information for Patient.

**Figure 3:**
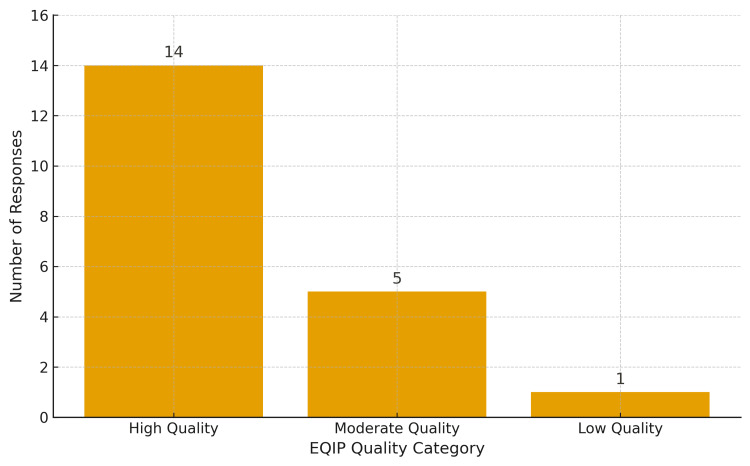
Categorization of ChatGPT responses based on EQIP quality ratings. Distribution of ChatGPT responses across quality categories according to the EQIP evaluation. The majority of responses (14 out of 20) were rated as high quality, five as moderate, and one as low quality. EQIP: Ensuring Quality Information for Patient.

As shown in Figure 2, EQIP scores were narrowly distributed between 80 and 90, with most responses clustering in the high-quality range. The categorical distribution of EQIP ratings is presented in Figure 3, illustrating that 14 responses were rated as high quality, 5 as moderate, and 1 as low quality.

Readability assessment

Readability assessments were conducted using three established indices: Flesch Reading Ease (FRE), Flesch-Kincaid Grade Level (FKGL), and Simple Measure of Gobbledygook (SMOG).

The mean scores were FRE = 57.8 ± 3.0, FKGL = 8.4 ± 0.6, and SMOG = 7.5 ± 0.5. These values indicate that ChatGPT’s responses were generally written at an 8th-9th grade reading level, which is acceptable for general audiences, though individuals with limited health literacy may still experience comprehension challenges.

**Figure 4:**
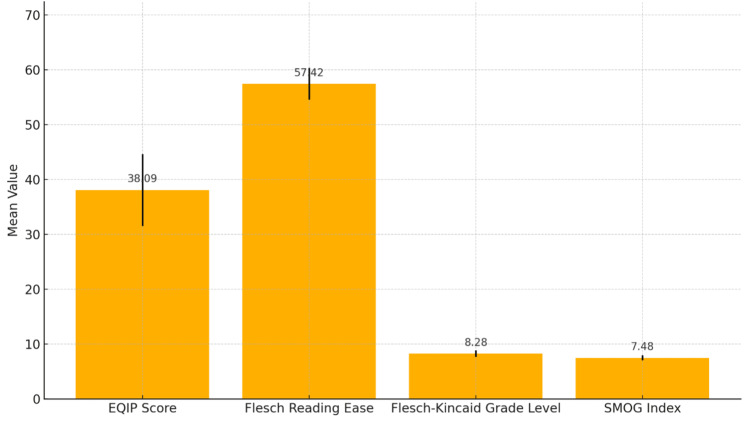
Combined distribution of EQIP quality scores and readability metrics (FRE, FKGL, SMOG) for ChatGPT responses. Mean values and standard deviations for EQIP, FRE, FKGL, and SMOG scores, summarizing both informational quality and linguistic complexity. FRE: Flesch Reading Ease; FKGL: Flesch-Kincaid Grade Level; SMOG: Simple Measure of Gobbledygook; EQIP: Ensuring Quality Information for Patient.

The detailed readability metrics for each query are presented in Table 2. To visualize the relationship between informational quality and linguistic complexity, Figure 4 integrates the EQIP scores with FRE, FKGL, and SMOG results, providing a consolidated overview.

**Table 2:** Readability metrics for ChatGPT responses to the 20 most frequently searched toothache-related queries. Readability results based on the Flesch Reading Ease (FRE), Flesch-Kincaid Grade Level (FKGL), and Simple Measure of Gobbledygook (SMOG) indices for ChatGPT responses.

Rank	Keyword	FRE	FKGL	SMOG
1	How to stop toothache	62.1	7.5	6.7
2	How to relieve toothache fast	59.5	7.9	7.2
3	Toothache remedies	55.8	8.2	7.5
4	Home remedies for toothache	61.9	7.6	6.9
5	Toothache at night	55.3	8.8	7.9
6	Toothache pain relief	58.2	8.2	7.5
7	Toothache causes	58.7	8.3	7.6
8	Toothache remedies instant	60.2	7.8	7.1
9	Toothache medicine	54.4	9.1	8
10	Severe toothache relief	57.9	8.2	7.5
11	Best painkiller for toothache	58.6	8.1	7.5
12	Toothache antibiotics	51.2	9.5	8.3
13	Wisdom toothache relief	59	8	7.6
14	Natural remedies for toothache	59.3	7.7	7.2
15	Best painkiller for severe toothache	52.3	9.4	8.2
16	Toothache remedies that work	61.1	7.4	6.8
17	Wisdom toothache	60	7.5	6.9
18	Toothache after filling	56.1	8.5	7.7
19	Toothache infection	55.6	8.6	7.9
20	Antibiotics for toothache	53.7	9.3	8.1

Qualitative observations

A qualitative review of ChatGPT’s responses revealed several notable features: frequent use of specialized dental terminology without simplified explanations, which may reduce accessibility for individuals with lower health literacy; occasional absence of explicit source attribution, which may undermine transparency and user trust; and clear structure and comprehensive coverage of topics, providing detailed information for users.

**Figure 5:**
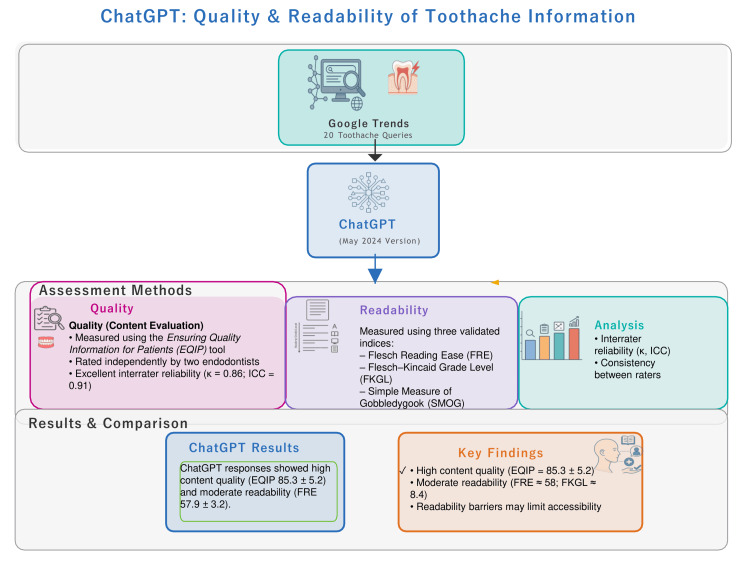
Visual summary of the study workflow and key analytical steps. The figure provides a visual summary of the study workflow and key analytical steps. It illustrates the process of data collection from Google Trends, response generation by ChatGPT, and subsequent quality (EQIP) and readability (FRE, FKGL, SMOG) assessments leading to the final key findings. FRE: Flesch Reading Ease; FKGL: Flesch-Kincaid Grade Level; SMOG: Simple Measure of Gobbledygook; EQIP: Ensuring Quality Information for Patient.

An overview of the study workflow and comparative findings is presented in Figure 5.

## Discussion

Key findings

This study evaluated the quality and readability of dental health information provided by ChatGPT in response to common toothache queries. ChatGPT consistently produced high-quality content, as indicated by elevated EQIP scores, yet readability analysis revealed only moderate accessibility. The coexistence of high content quality and limited readability raises a significant public health concern, as reliable information may remain inaccessible to individuals with lower health literacy, thereby limiting potential benefits and risking the reinforcement of health disparities.

Comparison with literature

This study evaluated the quality and readability of dental health information provided by ChatGPT in response to common toothache queries. ChatGPT consistently produced high-quality content, as indicated by elevated EQIP scores. However, readability analysis revealed only moderate accessibility. The coexistence of high content quality and limited readability raises a significant public health concern, as reliable information may remain inaccessible to individuals with lower health literacy. This may limit potential benefits and contribute to the reinforcement of health disparities.

These findings are consistent with prior research showing that even high-quality health information frequently poses readability challenges, reducing its effectiveness for populations with limited health literacy [26-29].

According to Nutbeam’s health literacy model and Norman and Skinner’s eHEALS framework, such linguistic complexity can hinder comprehension and contribute to digital health inequities [13].

Public health implications

Interpreted through Nutbeam’s model, the results suggest that ChatGPT responses may adequately support functional literacy (basic understanding of dental pain and management strategies), but their moderate readability limits the promotion of interactive literacy (applying advice to personal health situations) and critical literacy (evaluating credibility and making informed decisions) [30]. This gap may restrict the tool’s effectiveness in empowering users to make safe health-related choices.

Within the eHEALS framework, ChatGPT provides immediate and structured digital content, fulfilling the dimension of “access to online health information” [24]. However, the linguistic complexity revealed in readability scores may reduce users’ ability to critically evaluate or apply the information in real-life contexts [21]. These findings underscore the need for digital health communication strategies that not only deliver accurate content but also align with the diverse literacy capacities of users.

Strengths and limitations

A major strength of this study lies in its rigorous methodology, including systematic query selection via Google Trends and strong interrater reliability (Cohen’s κ = 0.86; ICC = 0.91). The multi-session approach enhanced reproducibility and minimized bias. However, several limitations should be acknowledged. Only English-language queries were included, which may limit generalizability across diverse populations. Another key limitation is the absence of direct user-centered testing. While readability indices provide standardized estimates of linguistic complexity, they cannot fully capture real-world comprehension. Future studies should therefore incorporate user feedback and comprehension testing, particularly among populations with diverse educational and health literacy backgrounds, to validate and extend the present findings.

Additionally, Google search results are subject to personalization and regional variability, which may influence comparator content despite the use of incognito mode. The study also relied on a single ChatGPT version (May 2024, collected in July 2024), which may introduce minor variability due to potential model updates. Finally, the analysis was restricted to Google Trends queries; future work should include patient-generated content from platforms such as YouTube or Reddit to capture broader information-seeking behaviors.

Future directions

Future studies should incorporate multilingual assessments, user-centered evaluations, and real-world comprehension testing to better capture the accessibility of AI-generated health information. In particular, engaging users with low or moderate health literacy will be essential to determine whether high-quality but complex AI outputs can be effectively understood and applied in practice. Comparative analyses across different AI platforms (e.g., ChatGPT versus other large language models) would also provide valuable insights. In addition, future research should explore policy frameworks and adaptive readability strategies to support equitable digital health communication on a global scale.

Such efforts will be essential not only for ensuring the safe and effective use of AI in dental health communication but also for promoting equity in digital health globally.

## Conclusions

This study revealed that ChatGPT provides high-quality information in response to the most frequently searched online queries about toothache; however, its readability remains at a moderate level. Although the content is generally accurate and comprehensive, complex sentence structures and the use of technical language may reduce accessibility for individuals with limited health literacy. The predominance of queries seeking immediate and self-manageable solutions highlights the public’s strong demand for rapid and comprehensible information. These findings are descriptive and exploratory in nature, reflecting the performance of a single model version within a defined period. Therefore, integrating simpler language, clearer medical terminology, and transparent source attribution into AI-generated outputs could enhance user trust and promote effective digital health communication. This approach aligns with Nutbeam’s health literacy model, which emphasizes the accessibility of health information across all literacy levels. Future research should investigate adaptive readability systems, multilingual evaluations, and real-world comprehension testing to further assess and improve the effectiveness of AI-driven health content across diverse populations.
